# Redirection to the bone marrow improves T cell persistence and antitumor functions

**DOI:** 10.1172/JCI97454

**Published:** 2018-04-09

**Authors:** Anjum B. Khan, Ben Carpenter, Pedro Santos e Sousa, Constandina Pospori, Reema Khorshed, James Griffin, Pedro Velica, Mathias Zech, Sara Ghorashian, Calum Forrest, Sharyn Thomas, Sara Gonzalez Anton, Maryam Ahmadi, Angelika Holler, Barry Flutter, Zaida Ramirez-Ortiz, Terry K. Means, Clare L. Bennett, Hans Stauss, Emma Morris, Cristina Lo Celso, Ronjon Chakraverty

**Affiliations:** 1 University College London (UCL) Cancer Institute, London, United Kingdom.; 2 UCL Institute of Immunity and Transplantation, London, United Kingdom.; 3 Department of Life Sciences, Imperial College London, London, United Kingdom.; 4 Center for Immunology and Inflammatory Diseases, Massachusetts General Hospital, Charlestown, Massachusetts, USA.; 5 The Francis Crick Institute, London, United Kingdom.

**Keywords:** Immunology, Therapeutics, Cancer immunotherapy, Chemokines, T cells

## Abstract

A key predictor for the success of gene-modified T cell therapies for cancer is the persistence of transferred cells in the patient. The propensity of less differentiated memory T cells to expand and survive efficiently has therefore made them attractive candidates for clinical application. We hypothesized that redirecting T cells to specialized niches in the BM that support memory differentiation would confer increased therapeutic efficacy. We show that overexpression of chemokine receptor CXCR4 in CD8^+^ T cells (T^CXCR4^) enhanced their migration toward vascular-associated CXCL12^+^ cells in the BM and increased their local engraftment. Increased access of T^CXCR4^ to the BM microenvironment induced IL-15–dependent homeostatic expansion and promoted the differentiation of memory precursor–like cells with low expression of programmed death-1, resistance to apoptosis, and a heightened capacity to generate polyfunctional cytokine-producing effector cells. Following transfer to lymphoma-bearing mice, T^CXCR4^ showed a greater capacity for effector expansion and better tumor protection, the latter being independent of changes in trafficking to the tumor bed or local out-competition of regulatory T cells. Thus, redirected homing of T cells to the BM confers increased memory differentiation and antitumor immunity, suggesting an innovative solution to increase the persistence and functions of therapeutic T cells.

## Introduction

The effector functions of T cells can be redirected against tumor antigens by gene transfer of T cell receptors (TCRs) or chimeric antigen receptors (CARs) to create large numbers of tumor-reactive cells for adoptive transfer. Early-phase clinical trials of antitumor T cells have shown significant efficacy in certain tumors (e.g., the use of anti-CD19 CAR-T cells for the treatment of acute lymphoblastic leukemia), and several parameters have emerged that predict response; these include the number of cells infused, their replicative potential, and their in vivo persistence following transfer (1–9). Host factors — for example, the peak levels of IL-15, a homeostatic cytokine that supports T cell proliferation and survival — also appear important (10, 11). In animal models, it has been shown that less differentiated T cells show greater in vivo expansion and survival than fully differentiated effector cells (12), a finding that relates to the former’s greater capacity for self-renewal. According to the progressive model of memory T cell formation, memory stem cells (Tscm)and central memory cells (Tcm) are less differentiated T cells that can divide to self-renew as well as generate more differentiated effector cells (13). There has therefore been interest in developing methods to generate and expand Tscm and Tcm in order to increase efficacy of therapeutic T cells. Current experimental strategies aim to transiently restrict terminal differentiation following T cell activation, for example by modifying use of metabolic pathways (14), activating Wnt–β-catenin (15), inhibiting Akt-mTOR (16, 17), or exposing cells to the homeostatic cytokines IL-15 and IL-7 (18).

An alternative approach is to identify and exploit anatomical niches that potentiate the expansion of Tscm and Tcm. The bone marrow (BM) is one site that may potentially contain such specialized microenvironments, and it has been hypothesized that, akin to hematopoietic stem cells, memory T cells may reside in distinct niches that support quiescence or proliferation and, critically, self-renewal (19). As early as 3 days after infection, memory precursor CD8^+^ T cells that are quiescent relative to the bulk effector population locate preferentially in the BM (20). In humans, a quiescent subset of memory T cells characterized by high ATP-binding cassette (ABC) transporter expression is also enriched in the BM (21). Recent studies have shown that exclusion from or displacement to the BM can have profound effects on the generation and/or survival of memory T cells. For example, Tcm are characterized by higher surface expression of CXCR4 than other T cell subsets and preferentially recirculate to the BM in response to CXCL12 (22). CXCR4 is required for integrin activation and memory CD8^+^ T cell adhesion in BM microvessels (22); in its absence, Tcm show a reduced frequency in BM and deficits in self-renewal in response to homeostatic signals, although effector responses following antigen challenge are largely intact (23). A corollary of this is that absence of CCR7 expression in memory T cells leads to their displacement from lymph nodes to the BM, where they undergo enhanced IL-15–dependent expansion (24). Although T cell–specific niche-organizing cells in the BM have not yet been identified at high resolution, memory T cells can be found in close proximity to cells producing IL-15 or VCAM-1–expressing stromal cells expressing other molecules that could be important in promoting their survival and/or proliferation, including IL-7 and 4-1BBL (25–27).

In this study, we tested the hypothesis that overexpression of CXCR4 in therapeutic CD8^+^ T cells would improve their functions and antitumor efficacy by directing them to memory niches in the BM. We demonstrate that CXCR4 overexpression redistributes engrafting cells to the BM, where they migrate toward CXCL12-expressing cells associated with the vasculature. Following antigen activation, increased access to IL-15 in the BM microenvironment drives enhanced expansion of the modified T cells and promotes their differentiation into early memory-like cells with greater antitumor functions. Thus, CXCR4 overexpression in therapeutic T cells is a potential platform technology to increase the persistence and function following adoptive transfer.

## Results

### Adoptively transferred CD8^+^ T cells overexpressing CXCR4 are preferentially recruited to the BM.

**Figure 1:**
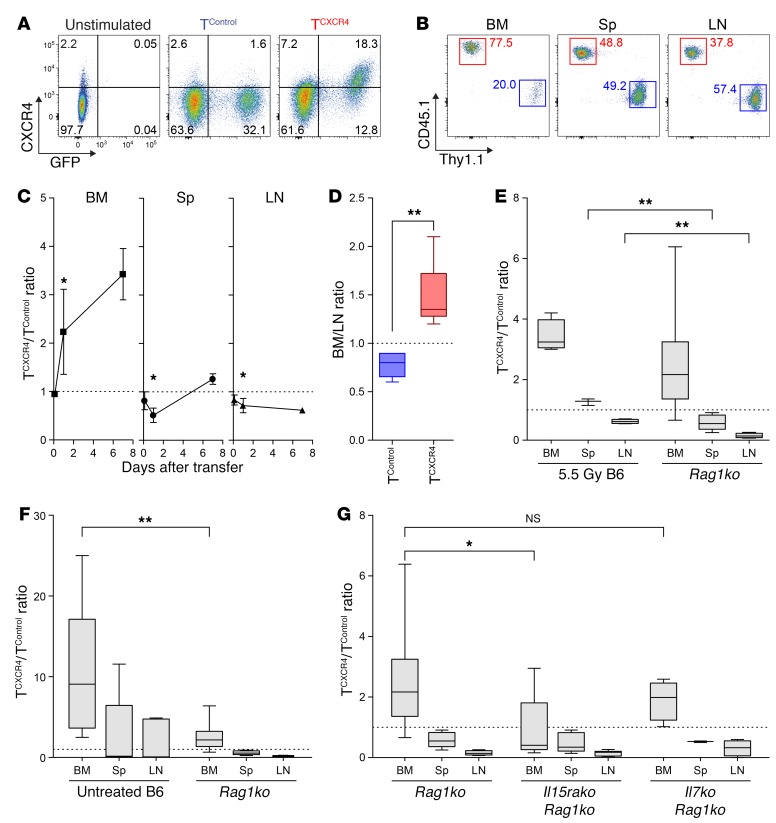
Adoptively transferred T^CXCR4^ demonstrate superior recruitment to the BM. (**A**) Representative flow cytometry plots for CXCR4 expression in untreated CD8^+^ T cells (unstimulated), T^Control^, or T^CXCR4^. Gating based on “fluorescence minus 1” controls. CXCR4 median fluorescence index (MFI): 380 unstimulated; 587 GFP^+^ T^Control^; 2,409 GFP^+^ T^CXCR4^. (**B** and **C**) Equal mixtures of T^CXCR4^ (CD45.1^+^) and T^Control^ (Thy1.1^+^) were injected into sublethally irradiated B6 mice. Representative plots of T^CXCR4^ (red) and T^Control^ (blue) frequencies in BM, spleen (Sp), and LN at day 7 are shown in **B**. Summary graphs in **C** indicate mean ± SD T^CXCR4^/T^Control^ ratio at timed intervals in BM, Sp, and LN (*n* = 6 per group at 3 and 24 hours, *n* = 4 per group at day 7). Statistical comparison was performed by Wilcoxon’s signed-rank test against a hypothetical ratio of 1.0 (dotted line). *P ≤ 0.05. (**D**) Box-and-whisker graphs for BM/LN ratio on day 14 following transfer of T^CXCR4^ or T^Control^ to separate sublethally irradiated B6 mice, calculated by division of percent GFP^+^ of BM CD8^+^ T cells by percent GFP^+^ of LN CD8^+^ T cells (*n* = 6 T^CXCR4^, *n* = 5 T^Control^). (**E**) Box-and-whisker graphs of T^CXCR4^/T^Control^ ratio in BM, Sp, and LN at day 7 following transfer into sublethally irradiated B6 mice (*n* = 4) and untreated *Rag1ko* mice (*n* = 10). (**F**) Box-and-whisker graphs of T^CXCR4^/T^Control^ ratio in BM, Sp, and LN at day 7 following transfer into untreated B6 mice (*n* = 11) and untreated *Rag1ko* mice (*n* = 10). (**G**) Box-and-whisker graphs of ratio of T^CXCR4^/T^Control^ in BM, Sp, and LN at day 7 following transfer into *Rag1ko* (*n* = 10), *Rag1ko.Il15rako* (*n* = 10), and *Rag1ko.Il7ko* (*n* = 4). Statistical comparisons in **D** and **E** were made using the Mann-Whitney test (2-tailed). *P ≤ 0.05, **P ≤ 0.01. All data are pooled from 2–3 independent experiments.

To determine whether increasing CXCR4 surface expression in murine T cells would influence their distribution, we transduced polyclonal CD8^+^ T cells from C57BL/6 (B6) mice with a modified pMP71 retroviral vector containing murine *Cxcr4* and *Gfp* reporter sequences (T^CXCR4^) or with a control vector containing *Gfp* alone (T^Control^). As shown in Figure 1A and Supplemental Figure 1 (supplemental material available online with this article; https://doi.org/10.1172/JCI97454DS1), both untreated CD8^+^ T cells and T^Control^ expressed a low level of cell surface CXCR4. Compared with GFP^+^ T^Control^, GFP^+^ T^CXCR4^ showed a median of 11.3-fold increase in CXCR4 expression (range 2.2–41.2, *P* = 0.03; Wilcoxon signed-rank test against a hypothetical ratio of 1.0). We then injected an equal mix of T^CXCR4^ (derived from B6 CD45.1 congenic mice) and T^Control^ (derived from B6 Thy1.1 mice) into B6 hosts (CD45.2, Thy1.2) receiving sublethal irradiation and used the respective congenic markers to measure the relative numbers of each transferred population in the BM, peripheral lymph node (LN), and spleen. At 3 hours, the initial engraftment of each transduced T cell population at each site was equivalent as indicated by a T^CXCR4^/T^Control^ ratio close to 1.0 (ratio 1.0 indicated by dotted line; Figure 1C). However, by 24 hours T^CXCR4^ accumulation in the BM was 2- to 3-fold greater than T^Control^ accumulation, whereas accumulation in the peripheral LN and spleen was moderately reduced. Seven days after transfer, the preferential redistribution of T^CXCR4^ to the BM had increased to 3- to 4-fold over controls (Figure 1, B and C). The pattern of increased distribution of T^CXCR4^ to the BM and away from the LN was also found under noncompetitive conditions in which each transduced T cell population was transferred to separate irradiated mice (Figure 1D). Because irradiation of the BM can disrupt the sinusoidal structure and increase local expression of CXCL12 (28) (*Cxcl12* mRNA expression is shown Supplemental Figure 2), we also examined whether T^CXCR4^ would similarly outcompete control cells in the BM of nonirradiated *Rag1*-knockout (*Rag1ko*) mice. As demonstrated in Figure 1E, T^CXCR4^ showed a similar propensity for BM accumulation on day 7 following transfer to B6 mice pretreated with 5.5 Gy total-body irradiation and untreated *Rag1ko* mice, indicating that the competitive advantage of T^CXCR4^ in the BM was independent of direct effects of irradiation. Irradiation did, however, have a minor but significant impact in mitigating the reduced relative accumulation of T^CXCR4^ in the spleen and LN.

We hypothesized that T cells entering the BM with greater efficiency would be able to better access local niche elements that promote their proliferation and/or survival. If greater competition for niche-related factors or cells contributed to the increased accumulation of T^CXCR4^ in the BM over control cells, we reasoned that such an advantage would be enhanced under conditions in which such factors were limiting. In the same series of experiments as shown in Figure 1B, we therefore compared the distribution of T^CXCR4^ and T^Control^ in competitive experiments under lymphocyte-replete (untreated B6 mice) versus lymphocyte-deficient (*Rag1ko* mice) conditions, the former condition exposing infused T cells to greater competition with endogenous T cells. These experiments showed that the ratio of T^CXCR4^/T^Control^ at day 7 following transfer was about 4-fold greater in the BM of nonirradiated WT compared with *Rag1ko* mice (Figure 1F). These data suggested that the skewed distribution of T^CXCR4^ to the BM was most pronounced under conditions in which CD8^+^ T cells have to compete with other lymphoid cells for niche factors or cells supporting their survival and/or proliferation. Because CD8^+^ T cells compete for homeostatic cytokines, we determined whether access to either IL-15 or IL-7 influenced the redistribution of T^CXCR4^ to the BM. In evaluating the role of IL-15, we used recipient mice that lacked expression of IL-15 receptor α (IL-15Rα); in this case, host cells are unable to trans-present IL-15 via cell surface IL-15Rα, leading to a functional deficiency of IL-15 (11). For these experiments, we therefore injected an equal mix of polyclonal B6 T^CXCR4^ (CD45.1) and T^Control^ (Thy1.1) to *Rag1ko* mice or *Rag1ko* mice lacking IL-7 or IL-15Rα before evaluating relative accumulation on day 7. As shown in Figure 1G, the capacity of T^CXCR4^ to outcompete T^Control^ in the BM was dependent on host expression of IL-15Rα, whereas the absence of IL-7 had no significant effect; the dependence on IL-15Rα was specific to the BM at this early time point with no differences observed between the groups in the LN or spleen.

### T^CXCR4^ show increased migration toward BM CXCL12^+^ cells.

**Figure 2:**
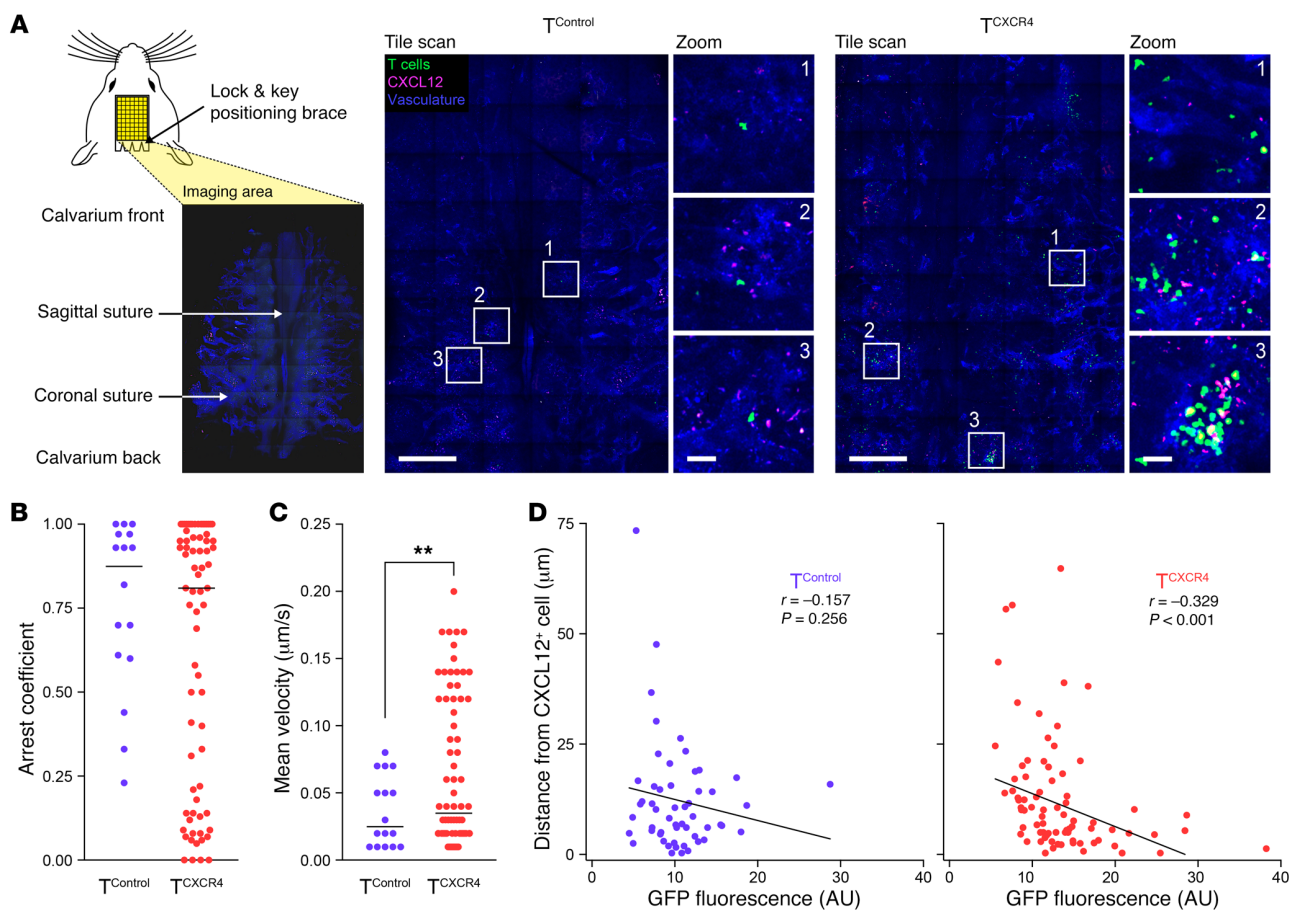
T^CXCR4^ show enhanced motility and directed migration to vascular-associated CXCL12^+^ cells in BM. (**A**) Left: Diagram showing strategy for calvarial imaging. Right: Intravital confocal calvarial imaging of transduced T cells (green) was performed 8 weeks after injection of T^Control^ and T^CXCR4^ into separate *Rag1ko* mice. Representative maximum projection tile scans and corresponding high-magnification insets are shown following i.v. injection of anti-CXCL12–PE (red) and Cy5-dextran to identify vasculature (blue). Scale bars: 500 μm in low-magnification images, 50 μm in inset images. (**B**) Summary graph showing arrest coefficient data for time-lapse imaging of T^Control^ and T^CXCR4^. Data are pooled from 5 mice (*n* = 2 T^Control^ and *n* = 3 T^CXCR4^). Median tracking period was 8.5 min/cell, range 8.5–30 min/cell (total number of cells tracked *n* = 72 T^CXCR4^, *n* = 16 T^Control^). (**C**) Summary graph showing mean velocity of tracked cells. Statistical comparison was made using a *t* test (2-tailed). **P ≤ 0.01. (**D**) *X*-*y* graphs showing GFP intensity (*x* axis) versus distance (*y* axis) of individual T^Control^ (*n* = 54, left) and T^CXCR4^ (*n* = 108, right) from CXCL12^+^ cells measured on static images derived from the same experiments in **A**–**C**. Inset to each graph shows Pearson’s correlation coefficient *r* and significance value.

To determine the respective behaviors of T^CXCR4^ versus T^Control^ in the BM, we performed intravital calvarial BM imaging following separate injection of transduced cells into nonirradiated *Rag1ko* mice; in each case, the GFP reporter was used to track the transduced cells. We used a tile-based imaging approach that permitted tissue-wide visualization of BM structures while maintaining a resolution permitting measurement of interactions with BM cells by time-lapse microscopy (Figure 2A). Recipient mice received i.v. injection of anti-CXCL12 phycoerythrin-conjugated (PE-conjugated) antibody 15 minutes before imaging to reveal CXCL12^+^ cells associated with the vasculature (identified by concurrent injection of Cy5-dextran). In separate experiments using *Flk1-Gfp* reporter mice to identify endothelial cells in relation to CXCL12 staining, we found that in vivo–labeled, vasculature-associated CXCL12^+^ cells were both Flk1^+^ and Flk1^–^ (Supplemental Figure 3). As shown in Figure 2A, T^CXCR4^ were far more numerous than T^Control^ and tended to be localized in loose clusters of cells rather than distributed evenly as individual cells throughout the BM space. To determine how CXCR4 overexpression affected the movement of T cells within the BM, we used time-lapse microscopy to track individual cells (Supplemental Videos 1–4). Cells moving very quickly through the circulation without adhering to the vasculature or entering the BM parenchyma were not included in our analysis. Frequently, cells within both the T^CXCR4^ and T^Control^ populations appeared to transiently scan the surface of vasculature-associated CXCL12^+^ cells. We also noted substantial heterogeneity in T^CXCR4^ behavior, with some cells moving very quickly in extravascular areas, whereas other cells with a rounder shape (cells sticking or rolling on the intraluminal surface of vessels or within the BM parenchyma) moved more slowly. To define whether cells were moving predominantly or “pausing,” we characterized T cell behavior according to their arrest coefficients (defined as the proportion of time a cell’s instantaneous velocity was ≤2 μm/min). The T^Control^ group showed a tendency toward cells with high arrest coefficients (that is, cells were moving more slowly than 2 μm/min for the majority of the time). In contrast, the T^CXCR4^ population showed a clear bimodal distribution for arrest coefficient with a significant subset of cells with lower values (Figure 2B). Consistent with the concept that T^CXCR4^ contained a subset of cells that were more motile than T^Control^, their mean velocity was higher than that of T^Control^ (mean velocity 3.9 ± 3.3 μm/min vs. 2.2 ± 1.6 μm/min, *P* = 0.004; Figure 2C). Increased motility of T^CXCR4^ compared with controls could potentially relate to higher chemokinesis and/or chemotaxis. To assess whether the increased motility of T^CXCR4^ was consistent with greater chemotaxis toward CXCL12-producing cells than T^Control^, we analyzed the tiled image data to measure the distance between GFP^+^ T cells from each group and vascular-associated CXCL12^+^ staining. However, when we compared the entire GFP^+^ T^CXCR4^ versus T^Control^ populations, we found no difference between the groups (the T^CXCR4^ median distance to nearest CXCL12^+^ cell was 6.9 μm [range 0.3–64.8], whereas the T^Control^ median distance was 8.5 μm [range 0.3–73.4], *P* = NS). We reasoned that migration of T^CXCR4^ and proximity to CXCL12-producing cells might depend on the level of CXCR4 expression by individual cells. We therefore re-evaluated cell position of T^CXCR4^ and T^Control^ populations according to the expression level of the GFP reporter (which is proportionate to CXCR4 in T^CXCR4^ cells; see Figure 1B). As shown in Figure 2D, there was an inverse correlation between distance to CXCL12^+^ cells and GFP expression for T^CXCR4^ but not for T^Control^. Thus, CXCR4 overexpression in CD8^+^ T cells enhances their motility and directed migration toward vascular-associated CXCL12^+^ cells in the BM.

### T^CXCR4^ show enhanced memory precursor differentiation and function.

**Figure 3:**
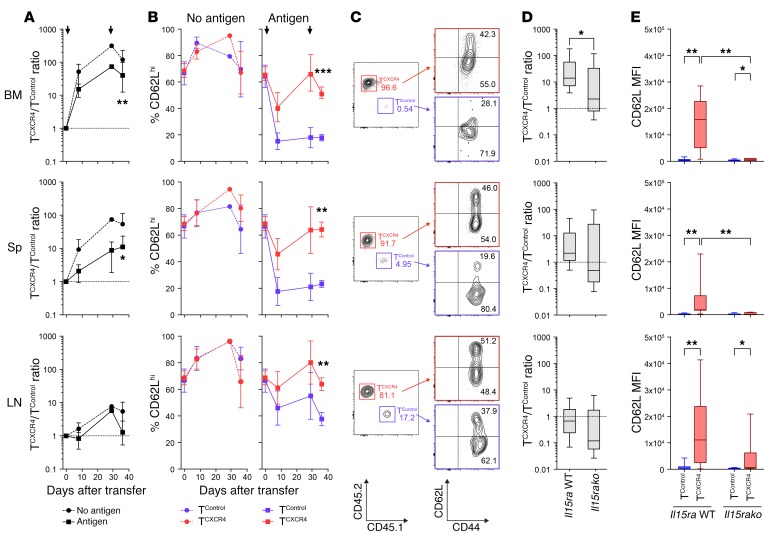
Ag-activated T^CXCR4^ retain a CD62L^hi^ phenotype. Equal numbers of OT-I T^CXCR4^ and T^Control^ were coinjected into *Rag1ko* mice, before prime-boost vaccination with relevant SIINFEKL peptide plus IFA (Antigen, *n* = 17) or irrelevant peptide plus IFA (No antigen, *n* = 8) on days 1 and 29. Tissues were harvested on day 8 (*n* = 4 per group), 29 (*n* = 3 Ag, *n* = 1 no Ag), and 36 (*n* = 9 Ag, *n* = 3 no Ag). Data from 4 independent experiments (with the exception of day 29 no-Ag group derived from 1 experiment). (**A**) Summary (mean ± SD) T^CXCR4^/T^Control^ in BM, Sp, and LN over time for no-Ag (circles, dashed lines) and Ag groups (squares, solid line). Arrows indicate time of prime-boost vaccination. (**B**) Summary (mean ± SD) CD62L expression over time in T^CXCR4^ (red) and T^Control^ (blue) in no-Ag (left) and Ag groups (right). (**C**) Representative plots for T^CXCR4^ and T^Control^ accumulation and surface expression of CD44 and CD62L on day 36 in BM, Sp, and LN. Numbers denote frequencies of T^CXCR4^ (red), T^Control^ (blue), and proportions of CD62L^hi^ and CD62L^lo^ cells (black). (**D**) Box-and-whisker graphs showing summary of T^CXCR4^/T^Control^ ratios in BM, Sp, and LN on day 36 following transfer into *Rag1ko* (*Il15ra* WT, *n* = 9) and *Rag1ko.Il15rako* (*Il15rako*, *n* = 6) mice undergoing the same prime-boost vaccination schedule outlined in **A**. Data derived from 3 independent experiments. (**E**) Box-and-whisker graphs showing summary data for CD62L expression by T^Control^ or T^CXCR4^ on day 36 in the same experiments outlined in **D**. In **A** and **D**, statistical comparisons were made by Wilcoxon’s signed-rank test against a hypothetical ratio of 1.0 (dotted line). *P ≤ 0.05, **P ≤ 0.01. In **B**, **C**, and **E**, statistical comparisons were made using the Mann-Whitney test (2-tailed). *P ≤ 0.05, **P ≤ 0.01, ***P ≤ 0.001.

To test how CXCR4 overexpression would affect the functions of antigen-activated (Ag-activated) CD8^+^ T cells, we transduced murine OT-I cells (from either a CD45.1 or a CD45.2 background) with the *Cxcr4* or control retroviral constructs and then transferred them at a 1:1 ratio to *Rag1ko* mice (Figure 3). To test responses to Ag, we used a prime-boost schedule in which a cohort of mice were vaccinated s.c. with ovalbumin peptide (SIINFEKL) and incomplete Freund’s adjuvant (IFA) on day +1 following transfer and again on day +29 (Ag^+^ group), whereas controls were vaccinated with irrelevant peptide and IFA (no-Ag group). As shown in Figure 3A, the OT-I T^CXCR4^/OT-I T^Control^ ratio in the no-Ag group showed an initial competitive advantage for OT-I T^CXCR4^ in the BM (day +8, mean ratio 51.5 ± 35.5). At later time points (day +28 and day +36), OT-I T^CXCR4^ had extensively outcompeted control cells in both the BM and spleen, whereas in the LN they were similar in number. The OT-I T^CXCR4^/OT-I T^Control^ ratios followed a similar pattern in the Ag^+^ group but with a nonsignificant trend for lower ratios than in the no-Ag group (day 36; in BM, mean ratio 119.4 ± 106.9 no Ag, 15.2 ± 6.5 Ag^+^; in spleen, mean ratio 53.8 ± 58.0 no Ag, 9.0 ± 15.0 Ag^+^). These data are therefore consistent with a model in which homeostatic rather than Ag-driven signals are responsible for the OT-I T^CXCR4^ outcompeting OT-I T^Control^ in the BM. The finding that the competitive advantage for OT-I T^CXCR4^ is initially observed only in the BM but then, later, extends to the spleen suggests a 2-phase model in which CXCR4 overexpression first increases T cell trafficking to the BM and, second, leads to the enhanced expansion and engraftment of cells that can recirculate to other lymphoid organs. Consistent with this concept, in an independent vaccination experiment, OT-I T^CXCR4^ had an initial competitive disadvantage in the peripheral blood for 28 days following vaccination (with a nadir at day 14, median ratio 0.6, range 0.3–0.8), before demonstrating a later competitive advantage at this location on day 90 (median ratio 2.9, range 1.7–6.4, in blood; median 39.8, range 37.1–130.5, in BM; Supplemental Figure 4).

We next asked whether overexpression of CXCR4 would confer additional phenotypic or functional properties to CD8^+^ T cells responding to Ag. We first evaluated transduced T cells in mice immunized in the Ag^+^ and no-Ag groups for surface expression of CD62L, a cell adhesion molecule that is shed upon effector differentiation. In response to Ag challenge, the majority of OT-I T^Control^ showed reduced CD62L expression in all compartments, but especially the BM and spleen, consistent with differentiation to effector or effector memory cells, whereas in the no-Ag group T^Control^ remained CD62L^hi^. In sharp contrast, although there was some reduction in CD62L expression in comparison with the no-Ag group, the OT-I T^CXCR4^ population from Ag-exposed mice retained greater CD62L expression than OT-I T^Control^ at all sites tested (Figure 3, B and C). For example, in the BM the mean proportion of OT-I T^CXCR4^ with a CD62L^hi^ phenotype at day 36 was 64% versus 23% for OT-I T^Control^. Although the proportion of CD62L^lo^ effector cells was lower for the OT-I T^CXCR4^ population than controls, their absolute number was in fact higher in the BM at day 36 and equivalent in the LN and spleen (Supplemental Figure 5), a finding consistent with the greater expansion of the entire population; these data indicate that OT-I T^CXCR4^ are proficient in generating effector-like cells but that there is a shift in the overall repertoire to include a greater frequency of less differentiated cells. We considered the possibility that the shift toward a CD62L^hi^ phenotype in Ag-activated OT-I T^CXCR4^ following the s.c. vaccination protocol we had used occurred as a result of their redistribution away from LN to the BM; in this circumstance, some T cells might receive less antigenic stimulation. Thus, we rerouted vaccination by using peptide-pulsed CD11c^+^ DCs intravenously, a method that seeds DCs to the spleen and BM but not LN (29). As shown in Supplemental Figure 6, OT-I T^CXCR4^ still had a competitive advantage in the BM and retained greater CD62L expression than OT-I T^Control^ in all compartments. To determine whether increased competition for an IL-15Rα^+^ niche in the BM could explain the greater expansion of CD62L^hi^ OT-I T^CXCR4^ in response to Ag, we compared the day 36 responses of OT-I T^CXCR4^ versus control cells in *Rag1ko*.*Il15ra* WT or *Rag1ko*.*Il15rako* recipient mice. As shown in Figure 3, D and E, the presence of IL-15α in the host was necessary for OT-I T^CXCR4^ to outcompete control cells in the BM, whereas this was not the case in the spleen or LN. The enhanced retention of CD62L expression by OT-I T^CXCR4^ following Ag exposure was also dependent on host IL-15α expression in the BM and spleen, with a similar trend in the LN. We noted slightly higher expression of CD62L by OT-I T^CXCR4^ in the BM and LN even in the absence of host IL-15α expression; this suggests that CD62L expression in OT-I T^CXCR4^ is also regulated to a minor extent by a mechanism independent of host IL-15α.

**Figure 4:**
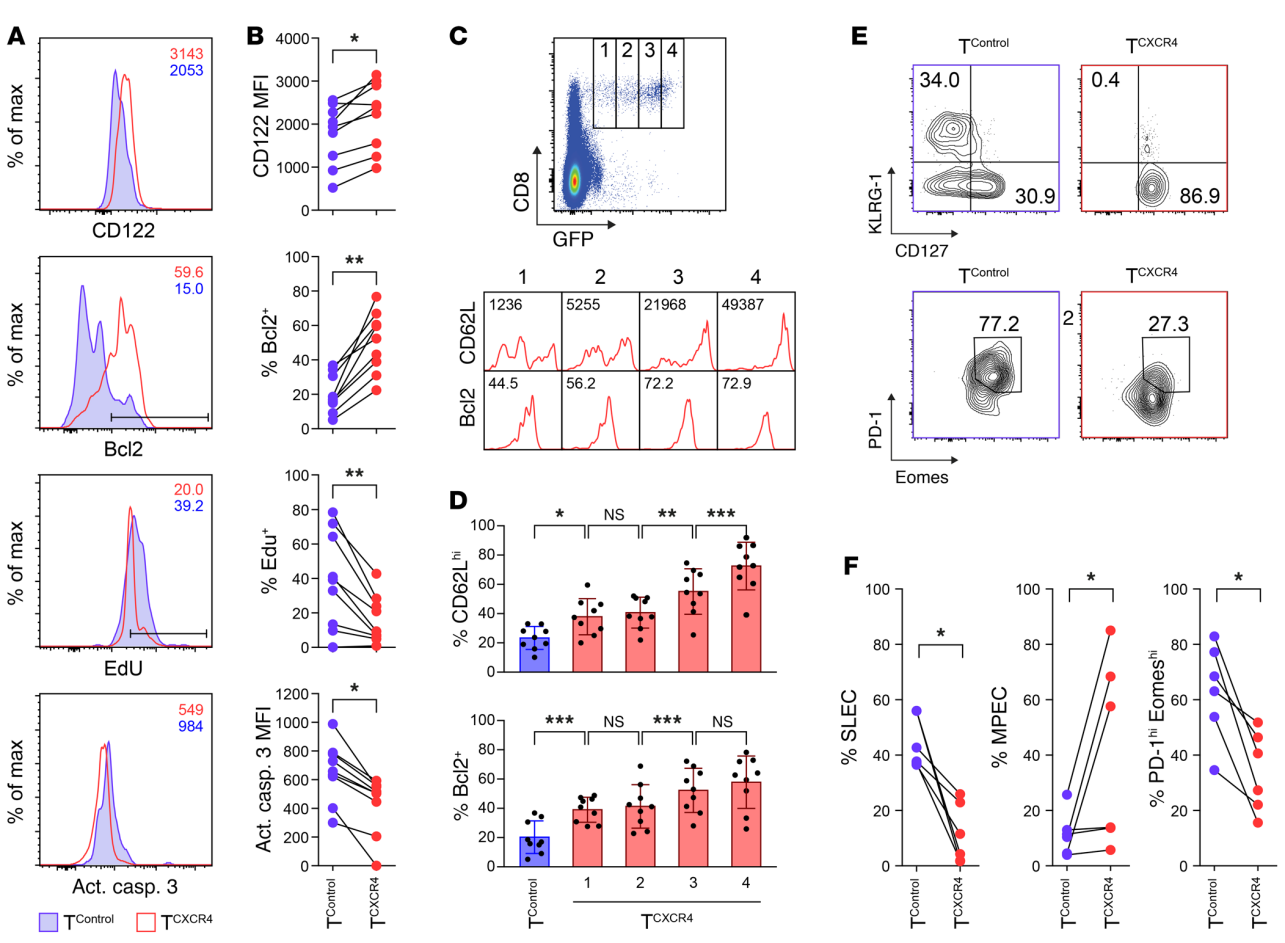
Ag-activated T^CXCR4^ adopt a less differentiated memory phenotype. Equal numbers of OT-I T^CXCR4^ and T^Control^ were coinjected into *Rag1ko* mice, which then underwent prime-boost vaccination with relevant SIINFEKL peptide plus IFA on days 1 and 29. Tissues were harvested on day 36 (*n* = 9); data are pooled from 4 independent experiments. (**A** and **B**) Representative flow cytometric histograms (**A**) and summary data (**B**) for expression of surface IL-15Rβ (CD122), intracellular Bcl2, EdU incorporation, and caspase-3 activity in T^Control^ (blue) versus T^CXCR4^ (red) on day 36 in cells isolated from the BM. Statistical significance was tested using Wilcoxon’s ranked-sum test (2-tailed). *P ≤ 0.05, **P ≤ 0.01. (**C** and **D**) Representative flow cytometric histograms (**C**) and summary data (mean ± SD) (**D**) for CD62L and Bcl2 staining in BM T^CXCR4^ gated according to GFP reporter expression (gates 1–4).Numbers shown as insets of the flow cytometric histograms relate to CD62L median fluorescence index (MFI) and proportion of Bcl2^+^ cells in the gated subset. Statistical significance was tested using the Mann-Whitney test (2-tailed). *P ≤ 0.05, **P ≤ 0.01, ***P ≤ 0.001. (**E** and **F**) Representative flow cytometric contour plots (**E**) and summary data (**F**) for frequency of splenic T^Control^ (blue) and T^CXCR4^ (red) with short-lived effector cell (SLEC) (KLRG-1^hi^CD127^lo^), MPEC (KLRG-1^lo^CD127^hi^), and exhausted (PD-1^hi^Eomes^hi^) phenotypes on day 36 (*n* = 5). Statistical significance was tested using Wilcoxon’s ranked-sum test (2-tailed). *P ≤ 0.05.

Because a CD62L^hi^ phenotype of Ag-activated T cells is associated with greater memory differentiation (13), we also evaluated Ag-activated OT-I T^CXCR4^ and T^Control^ in the BM for other phenotypic and functional properties that mark memory cells. At day 36 following vaccination, the OT-I T^CXCR4^ population was characterized by higher surface expression of IL-15Rβ (CD122), a receptor that is highly expressed on memory phenotype cells and is required for responsiveness to trans-presented IL-15 (11). In addition, OT-I T^CXCR4^ had higher intracellular expression of Bcl2, an antiapoptotic molecule that is also upregulated in memory cells (Figure 4, A and B). The CD62L^hi^Bcl2^hi^ profile was positively correlated with the level of CXCR4 expression as inferred from the intensity of GFP reporter fluorescence (Figure 4, C and D). To determine the proliferative response of transduced T cells following Ag exposure, we administered 5-ethynyl-2′-deoxyuridine (EdU) to mice by i.p. injection at timed intervals following vaccination and evaluated its incorporation 24 hours later. The proliferative burst of T cells in response to Ag was blunted in OT-I T^CXCR4^ compared with OT-I T^Control^ following both the prime and the boost vaccination (Supplemental Figure 7 and Figure 4, A and B). However, consistent with their greater expansion than control cells and higher expression of Bcl2, OT-I T^CXCR4^ had reduced levels of apoptosis as measured by staining for activated caspase-3 (Figure 4, A and B). Because these data were consistent with OT-I T^CXCR4^ possessing a “less differentiated” memory phenotype compared with controls (13), we also evaluated the expression of other markers that delineate different stages of differentiation in T cells responding to Ag. In T cells isolated from the spleen at day 36 (to allow a greater number of comparisons with control cells, which were limiting in the BM), the OT-I T^CXCR4^ population showed reduced frequencies of cells with a terminally differentiated state (Figure 4, E and F). Thus, compared with controls, OT-I T^CXCR4^ contained lower frequencies of cells that were KLRG-1^hi^CD127^lo^ (a phenotype of short-lived effector cells) but reciprocally higher frequencies of cells that were KLRG-1^lo^CD127^hi^ (memory precursor effector cells [MPECs]) (30). Furthermore, the OT-I T^CXCR4^ population contained fewer cells that were dual-positive for the coinhibitory receptor programmed death-1 (PD-1) and the T-box transcription factor eomesodermin, a profile that is associated with a terminally differentiated, exhausted state (31). The above phenotypic and functional differences between OT-I T^CXCR4^ and OT-I T^Control^ were observed only in Ag^+^ mice, with no significant differences observed in no-Ag controls (data not shown).

**Figure 5:**
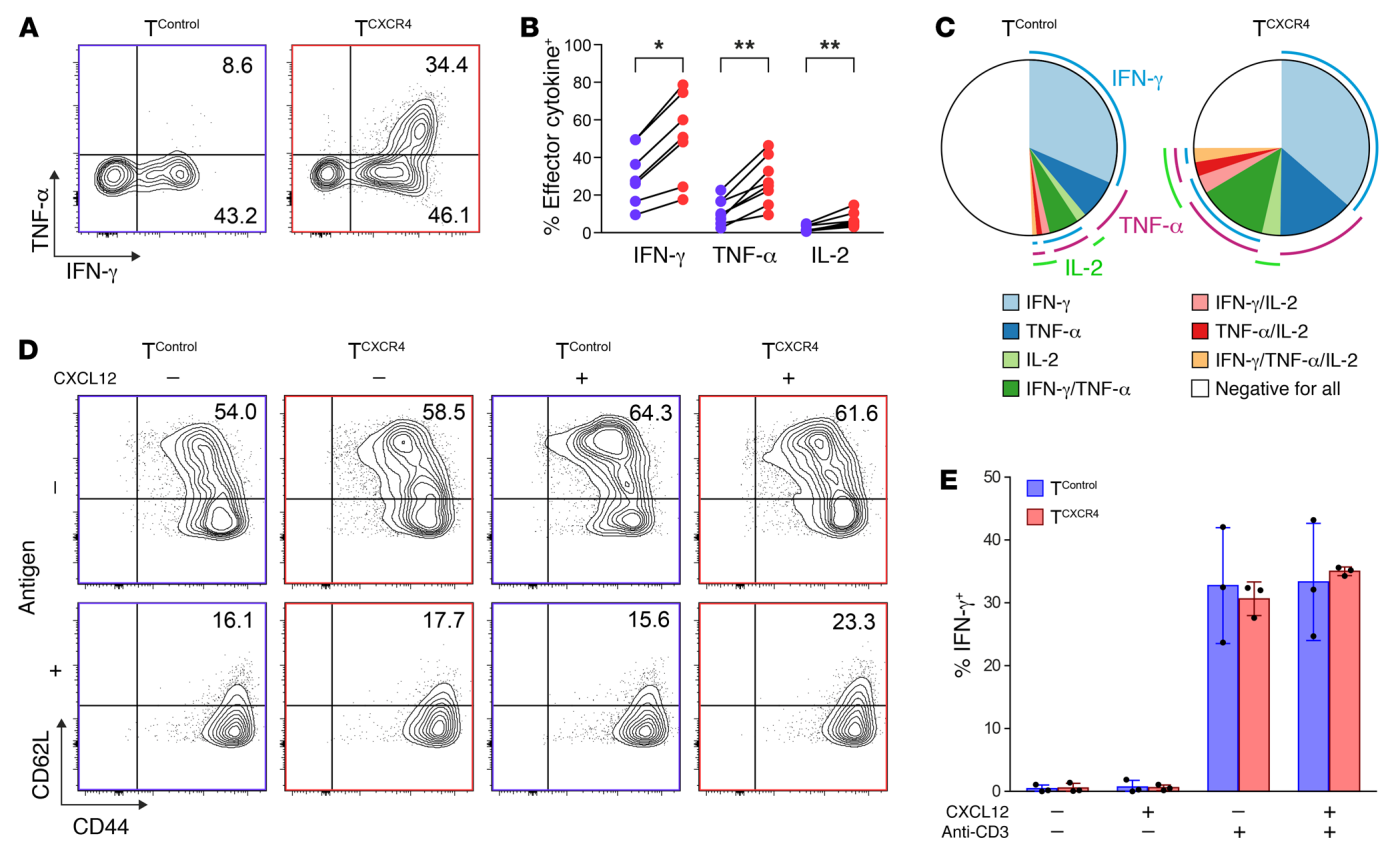
Ag-activated T^CXCR4^ have increased potential for polyfunctional cytokine generation. Equal numbers of OT-I T^CXCR4^ and OT-I T^Control^ were coinjected into *Rag1ko* mice, which then underwent prime-boost vaccination with relevant SIINFEKL peptide plus IFA on days 1 and 29. T cells were isolated from the spleen on day 36 (*n* = 7). (**A**) Representative flow cytometric contour plots showing IFN-γ and TNF-α intracellular costaining in OT-I T^CXCR4^ and OT-I T^Control^ after ex vivo stimulation with relevant peptide with gates set according to stimulation with irrelevant peptide. (**B**) Summary data for IFN-γ, TNF-α, and IL-2 generation from OT-I T^Control^ (blue) and OT-I T^CXCR4^ (red) in the same assays. Statistical significance tested using the Wilcoxon ranked sum test (two-tailed), *P ≤ 0.05, **P ≤ 0.01. (**C**) Pie charts depicting polyfunctional cytokine generation in T^Control^ and T^CXCR4^ according to Boolean combination gates identifying IFN-γ^+^, TNF‑α^+^, and IL-2^+^ cells. (**D**) Transduced OT-I T^CXCR4^ and OT-I T^Control^ were stimulated in vitro with relevant or irrelevant peptide and in the absence or presence of 500 ng/ml of recombinant murine CXCL12. Representative flow cytometric contour plots showing CD44 and CD62L surface expression. Data shown are representative of 2 independent experiments. (**E**) Summary data (mean ± SD) for intracellular IFN-γ generation after in vitro stimulation with anti-CD3 in the presence or absence of CXCL12 (*n* = 3 from 3 independent experiments).

To test the functional properties of transduced T cells, we isolated OT-I T^CXCR4^ and OT-I T^Control^ from the spleens of mice on day +36 and restimulated cells with relevant and irrelevant peptide over 4 hours ex vivo. OT-I T^CXCR4^ demonstrated enhanced effector IFN-γ, TNF-α, and IL-2 cytokine generation in response to ex vivo stimulation (Figure 5, A and B). Furthermore, by applying Boolean gating strategy, we detected an increase in the frequency of polyfunctional T cells among the OT-I T^CXCR4^ population (mean percentage of cells expressing 2 or more cytokines, 21.3% ± 6.2% for OT-I T^CXCR4^ vs. 8.7% ± 6.1% for OT-I T^Control^, *P* < 0.001; Figure 5C). Because CXCL12 binding is reported to amplify TCR signaling and function in T cells (32), we also determined the effect of exposure to recombinant CXCL12 on memory phenotype or cytokine generation of freshly transduced OT-I T^CXCR4^ and OT-I T^Control^ following TCR activation in vitro. As shown in Figure 5, D and E, CXCL12 had no effect on either CD62L expression or cytokine generation of OT-I T^CXCR4^ and OT-I T^Control^ populations. Similar results were observed following titration of the concentration of CXCL12. Taken together with the data shown in Figures 3 and 4, the discrepancy between in vitro and in vivo phenotype indicates that the effect of CXCR4 overexpression on T cells relates to how it affects migration and exposure to environmental cues.

### Resting memory T^CXCR4^ possess a less differentiated memory signature.

**Figure 6:**
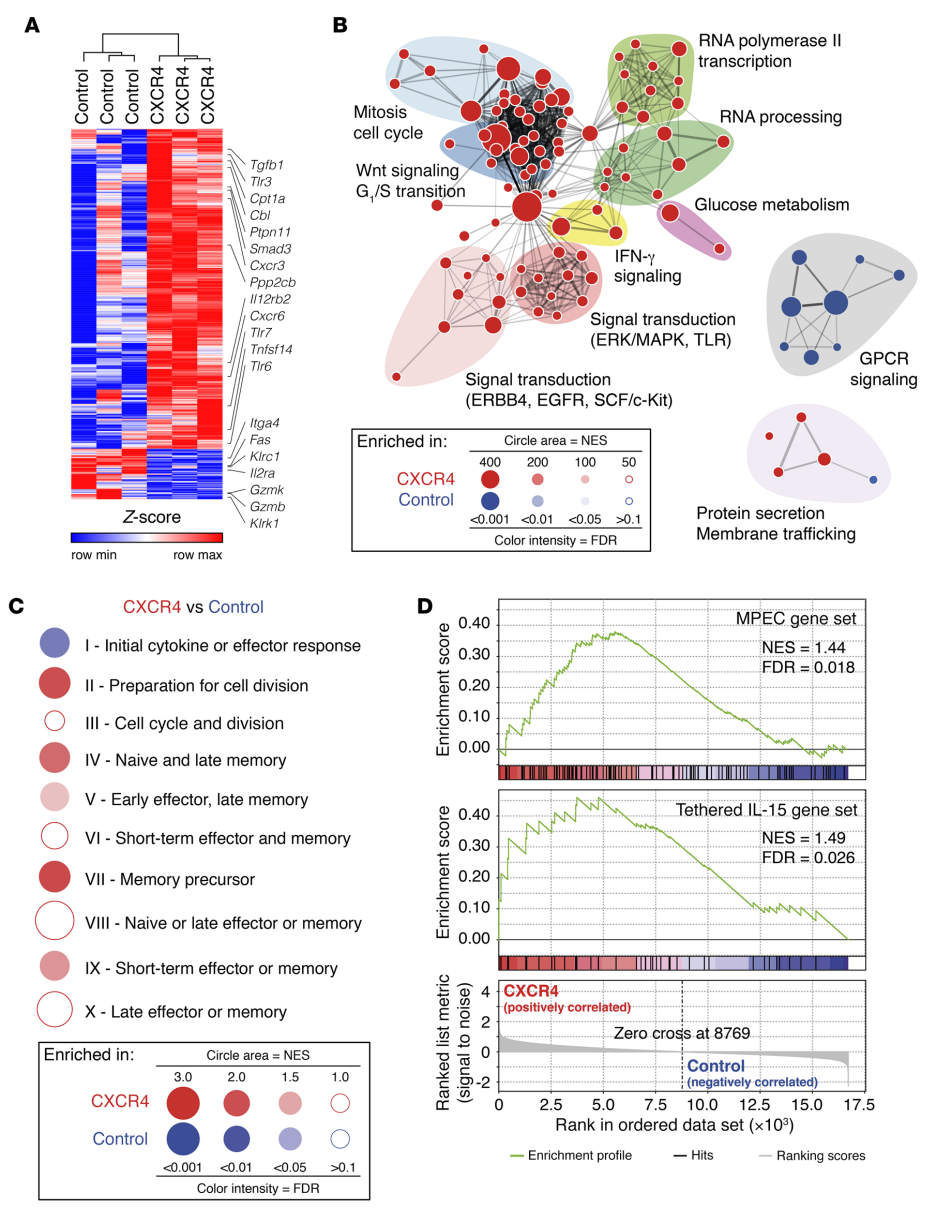
Resting memory T^CXCR4^ possess a memory precursor–like signature. (**A**) Heatmap showing the relative expression levels of the genes differentially expressed between resting memory OT-I T^CXCR4^ and controls (fold change ≥1.5, *P* ≤ 0.01); labels identify specific genes as discussed in the text. (**B**) The network map displays the Reactome gene sets enriched in OT-I T^CXCR4^ versus OT-I T^Control^. Node area indicates the size of the gene set; color code reflects enrichment in OT-I T^CXCR4^ (red) or OT-I T^Control^ (blue); color intensity is proportional to statistical significance. Clusters of functionally related gene sets were manually circled and assigned a label. NES, normalized enrichment score. (**C**) Gene set enrichment analysis–based (GSEA-based) assessment of the stage of antigenic response of OT-I T^CXCR4^ and OT-I T^Control^ according to the 10 phase-specific gene sets identified by Best et al. (42). Results are represented in a BubbleGUM plot, in which stronger and more significant enrichments are represented by larger and darker bubbles, colored red for OT-I T^CXCR4^ or blue for OT-I T^Control^. (**D**) GSEA plots showing that memory OT-I T^CXCR4^ upregulate genes associated with early memory cells and with increased responsiveness to IL-15.

To determine how enhanced CXCR4 expression affected memory T cell differentiation at the transcriptional level, we transferred OT-I T^CXCR4^ or OT-I T^Control^ to mice that were then vaccinated with peptide-pulsed DCs. Resting memory OT-I T^CXCR4^ or OT-I T^Control^ were then isolated from the spleen 90 days after DC vaccination, and gene expression profiling was performed. By this stage, OT-I T^CXCR4^ outnumbered controls at all sites (44.8 ± 40.1–fold in BM, 7.3 ± 3.1–fold in spleen, and 6.9 ± 3.3–fold in LN; data not shown) and had maintained a higher proportion of CD62L^hi^ cells at all sites (e.g., spleen, percentage CD62L^hi^ 93.2% ± 1.6% OT-I T^CXCR4^ vs. 73.5% ± 4.5% OT-I T^Control^; data not shown). Using a cutoff of ≥1.5-fold differential gene expression and a *P* value less than or equal to 0.01, 414 genes were upregulated in T^CXCR4^ compared with control cells and 63 genes were downregulated (Supplemental Table 1 and Figure 6A). Several genes upregulated in T^CXCR4^ or their related pathways have previously been implicated in promoting T cell memory differentiation or survival; these included *Cpt1a*, encoding an IL-15–regulated mitochondrial protein required for fatty acid oxidation in MPECs (33); the TNF superfamily ligand *Tnfsf14* (34); several genes associated with the Wnt pathway (e.g., *Ppp2cb*) (15) or TGF-β signaling (e.g., *Tggb1*, *Smad3*) (35); and the chemokine receptor genes *Cxcr3* (36) and *Cxcr6* (37). Other upregulated genes were linked to attenuation of TCR activation (e.g., *Ptpn11*, encoding the SHP2 phosphatase [ref. 38], and *Cbl*, encoding an E3 ubiquitin ligase [ref. 39]), a process that could be important in preventing terminal differentiation. The expression of several Toll-like receptor (TLR) genes was also increased in T^CXCR4^ (*Tlr3*, *Tlr6*, and *Tlr7*), although the role of direct TLR signaling in memory T cell function is less clear (40). Consistent with memory T^CXCR4^ maintaining a less differentiated state, genes with reduced expression compared with controls were those associated with cellular activation (e.g., *Ilr2a*, *Itga4*) and several genes encoding molecules associated with cellular cytotoxicity (e.g., *Klrk1*, *Klrc1*, *Gzmk*, *Gzmb*, and *Fas*). A gene set enrichment analysis using the Reactome database collection is represented in Figure 6B as an enrichment map, showing the pathways differentially enriched in T^CXCR4^ versus T^Control^ (see Supplemental Table 2 for complete list of enriched pathways). Memory T^CXCR4^ were enriched for gene sets relating to mitosis and cell cycle, metabolic functions, RNA processing and transcription, Wnt signaling, signal transduction (through MAPK/ERK, stem cell factor/Kit, EGFR, or TLR), and IFN-γ signaling. In contrast, gene sets related to signaling via G protein–coupled receptors (GPCRs) were downregulated in memory T^CXCR4^; since CXCR4 is a GPCR, this finding is consistent with receptor desensitization following continual stimulation (41).

To better align the transcriptional profiles of T^CXCR4^ to specific T cell differentiation states, we next compared our data with a published set of 10 gene clusters that segregate with specific phases of an in vivo OT-I response to Ag (42). As shown in the BubbleGUM plot (http://www.ciml.univ-mrs.fr/applications/BubbleGUM/index.html) in Figure 6C, T^CXCR4^ were primarily enriched for gene clusters II (preparation for cell division), IV (naive and late memory), and VII (memory precursors) as compared with controls. Notably, gene clusters IV and VII are also both enriched in MPECs that can be identified at the initiation of a CD8^+^ T cell response to Ag (30). As shown in Figure 6D, T^CXCR4^ showed enrichment for published gene signatures of MPECs identified on the basis of surface markers (CD127^hi^KLRG-1^lo^) (43). Finally, consistent with the requirement for IL-15 trans-presentation for the enhanced expansion of T^CXCR4^, we also found enrichment for a memory stem cell–like signature that is induced in human T cells gene-modified for constitutive signaling via a tethered IL-15–IL-15Rα complex (44) (Figure 6D).

### T^CXCR4^ demonstrate enhanced antitumor efficacy.

**Figure 7:**
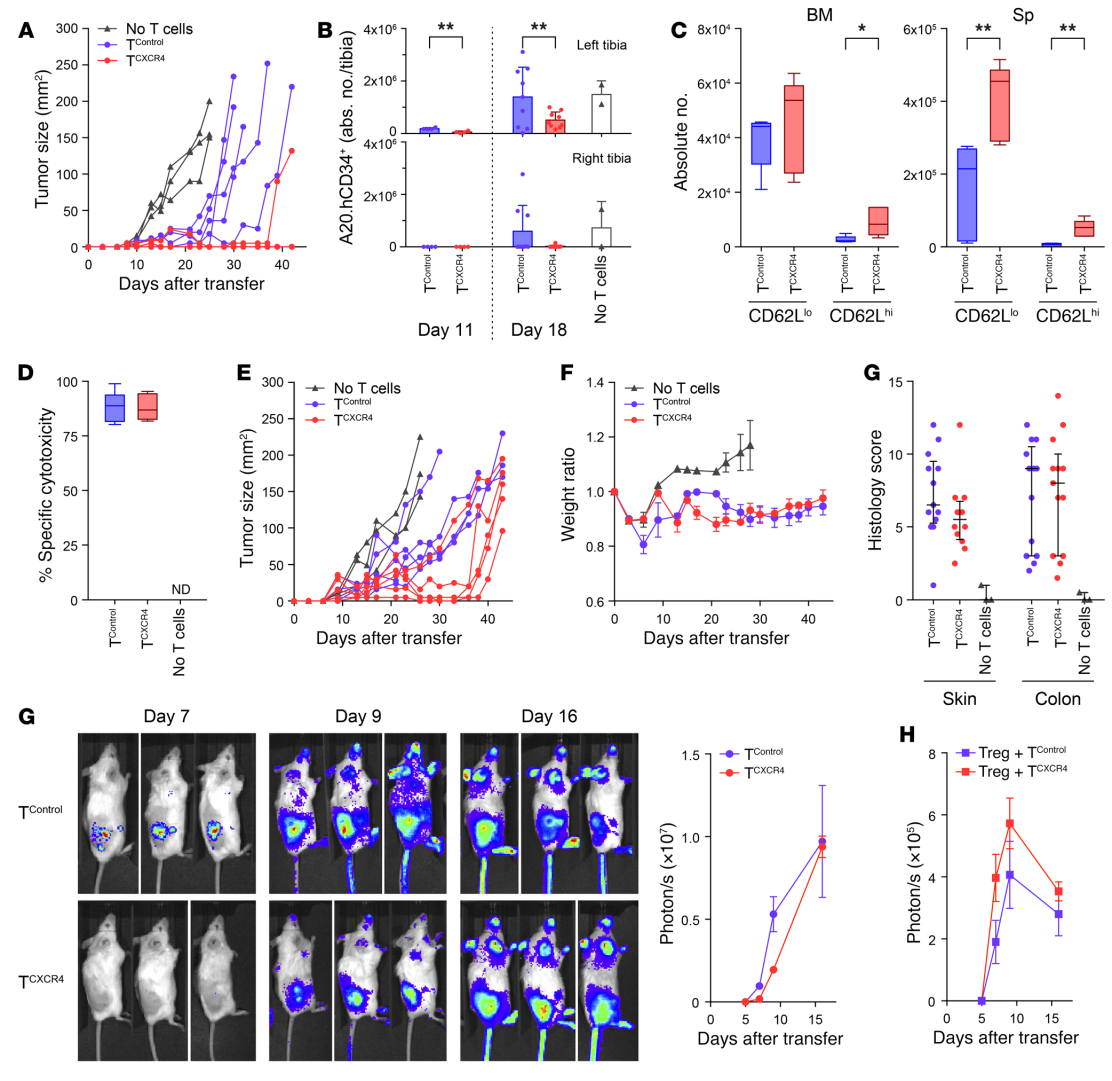
T^CXCR4^ demonstrate enhanced tumor protection. All mice underwent B6→BALB/c BMT. (**A**) A20 tumors were implanted on day 0; 2 days later, recipients received CD8^+^ allo-T^Control^ (blue circles, *n* = 5), CD8^+^ allo-T^CXCR4^ (red circles, *n* = 5), or no T cells (black triangles, *n* = 3). Graph shows tumor size at timed intervals. (**B**) A20.hCD34^+^ cells were given by intraosseous injection to the left tibia on day 0; 2 days later, recipients received CD8^+^ allo-T^Control^ (blue circles, *n* = 4 day 11, *n* = 9 day 18), 1 × 10^6^ CD8^+^ allo-T^CXCR4^ (red circles, *n* = 4 day 11, *n* = 9 day 18), or no T cells (black triangles, *n* = 2 day 18). Graphs show mean ± SD A20.hCD34^+^ accumulation in ipsilateral (left) and contralateral (right) tibia. Data pooled from 2 experiments. (**C**) CD8^+^ allo-T^Control^ or allo-T^CXCR4^ were given on day 2 (*n* = 5 per group); graph shows absolute numbers of CD62L^hi^ and CD62L^lo^ donor CD8^+^ T cells on day 10 post-BMT. *P ≤ 0.05, **P ≤ 0.01 Mann-Whitney test, 2-tailed. (**D**) Allo-T^Control^ and allo-T^CXCR4^ (*n* = 7 per group), or no T cells (*n* = 3), were given on day 2; graph shows in vivo specific cytotoxicity against BALB/c B cells on day 10 post-BMT. ND, no data. (**E**) A20 tumors were implanted s.c. on day 0; 2 days later, BMT recipients received no T cells (black triangles, *n* = 3), CD3^+^ allo-T^Control^ (blue circles, *n* = 5), or CD3^+^ allo-T^CXCR4^ (red circles, *n* = 5). Graph shows tumor size at timed intervals. (**F**) Experimental design as in **E**. Weight ratio and histological GVHD score on day 10 post-BMT (allo-T^Control^, *n* = 13; allo-T^CXCR4^, *n* = 13; no T cells, *n* = 3). Data pooled from 2 experiments. (**G**) On day 2, *luc^+^* CD3^+^ allo-T^Control^ or allo-T^CXCR4^ were transferred to BMT recipients bearing subcutaneous A20 tumors. T cell infiltration was monitored at timed intervals (mean ± SD, *n* = 3 per group). (**H**) Mean ± SD *luc^+^* Treg accumulation at timed intervals within A20 tumors following cotransfer on day 2 in a 1:1 ratio with non-*luc^+^* T^Control^ (blue squares) or T^CXCR4^ (red squares).

Taken together, the data in Figures 3–6 show that redirection of CD8^+^ T cells to the BM confers increased differentiation of memory precursor–like cells with greater potential for expansion and function. To examine how CXCR4 overexpression would impact on antitumor immunity, we evaluated graft-versus-tumor (GvT) responses mediated by allogeneic (B6, H-2^b^) T cells following transfer to tumor-bearing BALB/c (H-2^d^) recipients. Thus, BALB/c recipient mice received lethal irradiation followed on day 0 by i.v. infusion of T cell–depleted B6 BM and s.c. injection of 5 × 10^6^ host-strain B cell lymphoma, A20 (H-2^d^). Two days later, 1 × 10^6^ B6-derived CD8^+^ T^CXCR4^ or T^Control^ (allo-T^CXCR4^ or allo-T^Control^) were given i.v. to separate cohorts of tumor-bearing mice and the antitumor effects compared with controls not receiving T cells. Although both CD8^+^ allo-T^CXCR4^ and allo-T^Control^ exerted antitumor effects in allogeneic recipients compared with the no–T cell group, the efficacy of the T^CXCR4^ group was significantly greater than that of controls (Figure 7A; median survival >42 days vs. 31 days, *P* = 0.004 log-rank Mantel-Cox test). The enhanced antitumor efficacy of T^CXCR4^ compared with controls was dependent on alloreactivity, since no difference between the groups was observed in syngeneic mice (Supplemental Figure 8). Similar enhancement of T^CXCR4^ tumor control was observed when the above GvT model was adapted by switching of the site of tumor inoculation to unilateral left-sided intratibial injections of 5 × 10^5^ A20 cells (expressing human CD34 as a marker, huCD34.A20) before transfer, 2 days later, of 1 × 10^6^ CD8^+^ allo-T^CXCR4^ or allo-T^Control^ i.v., or no T cells. In the intraosseous injection model, allo-T^Control^ exerted no detectable control of local tumor growth in comparison with recipients receiving no T cells; in contrast, allo-T^CXCR4^ significantly delayed accumulation of A20 cells at this site (Figure 7B). We also observed a nonsignificant trend for a reduced frequency of tumor metastasis to the contralateral right tibia following allo-T^CXCR4^ infusion. To determine the phenotype of allo-T^CXCR4^ and allo-T^Control^ following adoptive transfer to allogeneic BM transplant (BMT) recipients, we determined the frequency and absolute number of CD62L^hi^ and CD62L^lo^ T cells in the BM and spleen at day 10. As expected, the majority of cells expanding in allo-T^Control^ repertoire were CD62L^lo^ effector T cells, with very low frequencies of CD62L^hi^ cells (median 4.7%, range 4.0%–13.7%, in BM, and 3.7%, range 2.9%–5.5%, in spleen). In contrast, although the majority of allo-T^CXCR4^ population also comprised CD62L^lo^ effectors, we observed greater retention of CD62L expression (median 15.4%, range 12.3%–21.0%, in BM, *P* < 0.05 vs. control, and 9.7%, range 8.8%–15.7% in spleen, *P* < 0.01 vs. control; Mann-Whitney 2-tailed). Notably, the absolute numbers of both CD62L^lo^ and CD62L^hi^ effector T cells were higher for allo-T^CXCR4^ than for allo-T^Control^ in spleen and equivalent in the BM (Figure 7C). These data indicate that transferred allo-T^CXCR4^ show a greater capacity for expansion of both effector cells as well as less differentiated memory precursor–like cells. Consistent with the capacity of allo-T^CXCR4^ to generate functional differentiated effector cells, specific cytotoxic responses against allogeneic target cells were similar in recipients of T^CXCR4^ and T^Control^ (Figure 7D).

We next considered the possibility that the enhanced fitness or expansion of allo-T^CXCR4^ could increase “on-target” toxicity by increasing the severity of graft-versus-host disease (GVHD). Because transfer of CD8^+^ without CD4^+^ allo-T cells is associated with only minor GVHD, we adapted the model by transferring 1 × 10^5^ CD3^+^ allo-T^CXCR4^ or allo-T^Control^ i.v. on day +2. As for CD8^+^ T cells alone, CD3^+^ allo-T^CXCR4^ showed greater antitumor activity than control cells (Figure 7E; median survival 43 days vs. 38 days, *P* = 0.01 by log-rank Mantel-Cox test), but this was not associated with increased GVHD severity as evidenced by equivalent weight loss in BMT recipients of allo-T^CXCR4^ and allo-T^Control^, and no increase in histological GVHD (Figure 7F).

Because B cell lymphomas are enriched for CXCL12-expressing stromal cells (45), it was possible that the greater efficacy of allo-T^CXCR4^ in comparison with controls could be linked directly to their increased recruitment to the tumor bed. We therefore repeated the GvT experiments but used CD3^+^ T cells from B6 firefly luciferase transgenic (*luc^+^*) mice to monitor the recruitment of allo-T^CXCR4^ versus allo-T^Control^. Following transfer of *luc^+^* CD3^+^ allo-T^CXCR4^ or allo-T^Control^ to allogeneic recipients bearing A20 tumors, the bioluminescent signals detected from the tumor site over the next 16 days were similar in both groups, albeit with a small delay in initial recruitment of allo-T^CXCR4^, indicating that the greater antitumor effects of T^CXCR4^ could not be explained by enhanced accumulation (Figure 7G). We also tested the possibility that allo-T^CXCR4^ could outcompete Tregs for limiting CXCL12-dependent niches within the tumor (45), by adapting the experimental design and cotransferring donor-strain *luc^+^* Tregs at a 1:1 ratio with either T^CXCR4^ or T^Control^ (both from B6 non-*luc^+^* donors). As shown in Figure 7H, Treg recruitment to the tumor site was similar in recipients receiving either T^CXCR4^ or T^Control^. Together, these data refute the hypotheses that enhanced accumulation of T^CXCR4^ and/or out-competition of Tregs in the tumor underpins their increased efficacy. Instead, transferred T^CXCR4^ have greater per-cell functions that translate into greater antitumor immunity.

## Discussion

We have demonstrated that redirection of therapeutic T cells to the BM confers a superior potential for expansion in vivo. Upon entering the BM sinusoids, T^CXCR4^ showed directed migration toward vascular-associated CXCL12^+^ cells and more efficiently competed for niches formed by IL-15Rα^+^ cells. Following immunization, T^CXCR4^ adopted a less differentiated memory program characterized by resistance to apoptosis, low expression of PD-1, and polyfunctional cytokine secretion; collectively these properties translated into a greater capacity for expansion and improved per-cell functions. This new strategy could potentially be used across multiple therapeutic platforms to improve engraftment, persistence, and functions of adoptively transferred T cells.

We found that greater competition for an IL-15Rα^+^–dependent niche in the BM underpins the superior homeostatic expansion of CD62L^hi^ T^CXCR4^ compared with control cells. These findings suggest that T^CXCR4^ can preferentially occupy a BM niche or niches that can be saturated, which is in keeping with the known capacity of endogenous memory T cells to restrict BM seeding of adoptively transferred memory T cells (46). Although cellular and other molecular components of an IL-15α^+^–dependent memory T cell niche remain to be determined, we considered whether binding to CXCL12 could directly influence memory differentiation independent of other niche elements. Recent studies have shown that CXCL12 stimulates the physical association between CXCR4 and the TCR within the immunological synapse, enhancing the recruitment and phosphorylation of multiple adaptor proteins (32). In doing so, CXCL12 amplifies the downstream intracellular signaling apparatus of the TCR (e.g., MAPK and AP-1 transcriptional activity), leading to increased proliferation and cytokine secretion (32). Furthermore, by promoting degradation of Bcl2-interacting mediator of death extra-long isoform (Bim_EL_), CXCL12 costimulation enhances T cell survival and promotes memory formation of CD4^+^ T cells. In this study, in vitro exposure of Ag-activated T^CXCR4^ to CXCL12 did not recapitulate the phenotypic or functional properties of T^CXCR4^ in vivo. However, some effects of CXCL12-CXCR4 interaction may not be easily reproduced in vitro, for example, the modulation of T cell adhesion to the extracellular matrix and/or guidance of cells toward other niche elements that act in concert. In addition, it is also possible that the capacity of CXCR4 to form heteromers with other GPCRs can permit cooperative functions with other ligands, independent of CXCL12 (47).

Three months after antigenic exposure, the transcriptional profiles of resting memory T^CXCR4^ aligned preferentially with less differentiated memory cells and included a number of genes known to be required for optimal memory generation (15, 33–37). In contrast to our findings following acute Ag exposure, which showed an initial blunting of the proliferative response, memory T^CXCR4^ were enriched for gene sets relating to cell cycle and mitosis, suggesting proliferative fitness in response to homeostatic cytokines. Although T^CXCR4^ demonstrated some features consistent with the phenotype of Tscm (15, 48) (e.g., a CD122^hi^Bcl2^hi^ phenotype, retention of CD62L upon antigenic challenge, and enrichment for gene sets linked to IL-15 and Wnt signaling), they were uniformly CD44^hi^, and expression of the stem cell marker Sca-1 was equivalent to that in control T cells (as opposed to the CD44^lo^Sca-1^hi^ phenotype of putative murine Tscm; data not shown). Because gene expression analysis was not performed at a single-cell level and was confined to splenic T cells (in the absence of sufficient T^Control^ in the BM for comparison), it is possible that resting memory T^CXCR4^ are in fact heterogeneous at the population level and that our analysis has missed more quiescent cells present as a minority population or at specific locations not sampled. In this scenario, T cells undergoing homeostatic proliferation outnumber more quiescent Tscm-like cells that undergo infrequent cell divisions. In this regard, quiescent, ABC transporter–expressing memory T cells can be mobilized from the human BM using a small-molecular inhibitor of CXCR4-CXCL12 interactions, suggesting that CXCR4-expressing quiescent T cell populations may normally exist at this site (21). These data reflect current limitations in defining core signatures for less differentiated memory T cells, including Tscm, and may require application of single-cell approaches to elucidate how repositioning of T cells to the BM ultimately influences cell fate.

Despite initial preferential redirection to the BM, T^CXCR4^ demonstrated the potential to traffic to other lymphoid tissues and showed equivalence in accumulation within subcutaneous tumors and peripheral tissues during GVHD. These findings refute the potential concern that T^CXCR4^ would be trapped within the BM, unable to egress to sites required for therapeutic efficacy. Consistent with downregulation of CXCR4 on transduced T cells upon transfer in vivo compared with input cells (data not shown), we found that gene expression signatures for GPCR signaling were in fact reduced in memory T^CXCR4^. These findings suggest that the overexpressed CXCR4 protein is still subject to desensitization following agonist binding via the physiological mechanisms of receptor internalization and ubiquitin-mediated degradation (49). This process may enable redeployment of T^CXCR4^ to sites other than the BM according to the precise stimulus in question and the expression of alternative homing receptors. Furthermore, the relative deficit in accumulation of T^CXCR4^ in the spleen and LN as cells initially redistributed to the BM was only transient, indicating that enhanced homeostatic expansion also has the potential to permit access of transduced T cells to other sites.

We excluded the possibility that increased antitumor efficacy of T^CXCR4^ related to enhanced trafficking to the tumor site. In response to hypoxia, stromal cells contained within organized tumors increase secretion of CXCL12, leading to enhanced tumor survival, neovasculogenesis, and recruitment of regulatory populations (50). The lack of increased accumulation of T^CXCR4^ compared with controls remained true even after correction for tumor size, which was different in the groups, or after transfer of larger numbers of T cells to exclude issues relating to the detection threshold of bioluminescent imaging (data not shown). Although higher expression of CXCL12 by stromal cells is reported in A20 and other transplantable B cell tumors (45), a number of other factors (e.g., expression of other homing receptors, in situ proliferation) may influence the overall accumulation signal. Notwithstanding these factors, our data indicate increased per-cell functions of transferred T^CXCR4^ compared with control cells. This conclusion therefore raises the question of why allo-T^CXCR4^ did not cause greater GVHD than allo-T^Control^. One potential explanation is suggested by the less differentiated phenotype of T^CXCR4^ following challenge with both nominal antigen and alloantigen; such cells may therefore lack key effector molecules associated with trafficking or cytotoxic functions. Indeed, resting memory T^CXCR4^ lacked gene expression encoding several molecules such as *Itga4* (51) and *Gzmb* (52) that are required for GVHD development. A non–mutually exclusive mechanism is that although T^CXCR4^ are able to egress from the BM, there is still an overall bias of these cells to recirculate to CXCL12-rich sites. Although CXCL12 is increased in the BM after irradiation and allo-BMT, CXCL12 expression levels in GVHD target organs remain relatively low (data not shown).

In conclusion, we have demonstrated that engineering of T cells to redirect them to the BM via CXCR4-CXCL12 increases their expansion and function. This innovative approach has the potential for multiple applications where the therapeutic efficacy requires long-term survival of functional T cells, for example in cancer or infectious disease. Further work to define the composition of the memory T cell niche in the BM in both health and disease may permit future refinement of the strategy.

## Methods

### Mice.

C57BL/6 and BALB/c mice were purchased from Charles River Laboratories. *B6.PL‑Thy1a/CyJ* (B6 Thy1.1), *B6.SJL-Ptprca Pepcb/BoyJ* (B6 CD45.1), *Flk1-Gfp*, and *Rag1ko* OT-I mice were purchased from The Jackson Laboratory and bred in house. Thy1.1^+^ C57BL/6 luciferase^+^ transgenic mice were a gift from Robert Zeiser (Freiburg University, Freiburg, Germany). *Rag1ko*, *Rag1ko.Il7ko*, and *Rag1ko.Il15rako* mice were a gift from Benedict Seddon (UCL, London, United Kingdom). UCL Biological Services bred the above mice in house; irradiated or immune-deficient recipients were maintained in individual ventilated cages. Animals used as recipients for BMT were 10–20 weeks old, and donors were 8–16 weeks old.

### Cell lines.

Ecotropic Phoenix packaging cells, used for retroviral particle production, were a gift from G.P. Nolan (Stanford University, Stanford, California, USA). The murine A20 B-lymphoblastic cell line has previously been described (53); in some experiments, the cell line was modified by pMP71 HuCD34 retroviral transduction to express human CD34 (A20.hCD34^+^) followed by isolation and sequential immunomagnetic enrichment using a human CD34 microbead kit (Miltenyi Biotec, Germany).

### Retroviral vectors and transduction.

The murine *Cxcr4* gene was subcloned into a pMP71 to generate pMP71-*Cxcr4*-IRES-*Gfp*. pMP71-IRES-*Gfp* was used as a control vector. Retroviral transduction was performed as described previously (54).

### Irradiation and BM transplantation.

For sublethal irradiation, mice received 5.5 Gy TBI. BMT was performed as described previously (55) with some modifications. Briefly, BALB/c mice (or B6 mice for syngeneic BMT controls) received lethal irradiation (4 Gy twice over 72 hours) before i.v. infusion of 5 × 10^6^ T cell–depleted B6 BM. On day 2 following BMT, mice were inoculated with 5 × 10^5^ A20 cells s.c. into the flank or with 5 × 10^5^ A20.hCD34^+^ by intra-bone injection into the left tibial BM cavity during anesthesia. Tumors were allowed to grow for 2 days before i.v. injection of B6 allo-T^CXCR4^ or allo-T^Control^. Mice were scored for clinical severity and weight 3 times per week or more frequently if necessary, and were sacrificed according to a predefined severity endpoint, or if tumor surface area was greater than 200 mm^2^ or developed surface ulceration. For tumor survival experiments, tumors greater than 150 mm^2^ were categorized as an event. Bioluminescent imaging of firefly luciferase^+^ T cell infiltration of tumors was performed as described previously (54).

### Prime-boost vaccination.

Isolation of CD8^+^ T cells or CD11c^+^ DCs was performed by immunomagnetic selection from splenocytes using Manual MACS Cell Separation Technology (QuadroMACS Separator, LS columns, CD8a [Ly-2] and CD11c MicroBeads; Miltenyi Biotec), according to the manufacturer’s instructions. For in vitro experiments, SIINFEKL peptide (Invitrogen) was added at a concentration of 5 μM. In competitive in vivo experiments, 5 × 10^5^ to 1 × 10^6^ each of T^CXCR4^ and T^Control^ were mixed into a 1:1 ratio before injection, and vaccination was performed at 24 hours and on day 29 by s.c. injection of 200 μM SIINFEKL or an irrelevant peptide in a 1:1 ratio with incomplete Freund’s adjuvant (Sigma-Aldrich). Alternatively, mice received 1 × 10^6^ CD11c^+^ peptide-loaded DCs intravenously.

### Isolation of murine immune cells.

To prepare cell suspensions from spleens and lymph nodes, the freshly removed organs were mashed and passed through a 40-μm cell strainer; red blood cells were removed by isotonic lysis with ammonium chloride (ACK Lysing Buffer; Lonza). Cells were resuspended in FACS buffer (PBS, 2% FCS, 2 mM EDTA; Lonza) for counting and immunolabeling. To isolate BM cells, both epiphyses of the long bones of the hind limbs were cut, and the BM was flushed out with FACS buffer. The cell suspension was filtered through a 40-μm cell strainer, and red blood cells were removed by isotonic lysis with ammonium chloride. Cells were resuspended in FACS buffer for counting and immunolabeling.

### Flow cytometry.

The following monoclonal antibodies were used for flow cytometry: anti–murine CXCR4 (clone 2B11), CD8a (clone 53-6.7), CD62L (clone MEL-14), CD127 (clone A7R34), CD122 (clone TM-β1), CD132 (clone TUGm2), CD45.2 (clone 104), TNF-α (clone MP6-XT22), IL-2 (clone JES6-5H4), Bcl2 (clone 3F11), and active caspase-3 (clone C92-605) (all supplied by BD Biosciences); and anti–murine Thy1.1 (clone H1S51), CD44 (clone IM7), CD25 (clone PC61), CD45.1 (clone A20), IFN-γ (clone XMG1.2), Eomes (clone Dan11mag), PD-1 (clone RMP1-30), and KLRG-1 (clone 2F1) (all supplied by eBioscience). For intracellular staining, cells were fixed and permeabilized with BD Cytofix/Cytoperm (BD Biosciences). For measurement of proliferation, animals were injected with 100 μg 5-ethynyl-2-deoxyuridine (EdU) i.p., and cells were subsequently stained using the Click-iT Assay Kit (Thermo Fisher Scientific). Multicolor flow cytometry data acquisition was done with BD LSRFortessa and BD LSR II cell analyzers equipped with BD FACSDiva v6.2 software (BD Biosciences). FACS was performed on a BD FACSAria equipped with BD FACSDiva v5.0.3 software (BD Biosciences). All samples were maintained at 4°C for the duration of the sort. A minimum of 5,000 cells were collected, and only those with purity ≥95% were used for RNA extraction. Cells were sorted directly into Buffer RLT (QIAGEN) with 1% 2-β-mercaptoethanol (Sigma-Aldrich), disrupted by vortexing at 3,200 rpm for 1 minute, and immediately stored at –80°C until further processing. Flow cytometry data were analyzed with FlowJo X v10 (FlowJo LLC).

### Evaluation of T cell function.

In vitro and ex vivo analysis of cytokine generation following peptide stimulation was performed as described previously (56). In vivo analysis of specific cytotoxicity was performed as described previously (55).

### Histological evaluation.

Histological evaluation of GVHD in the skin, gut, and liver was performed single-blinded following the scoring system previously described (55).

### Imaging.

Intravital microscopy was performed using a combined Zeiss LSM 780 upright confocal/2-photon microscope as described previously (57). Blood vessels were highlighted by i.v. injection of 8 mg/ml 500-kDa Cy5-dextran (Nanocs). The following antibodies were used: anti-CXCL12 (R&D Systems) and anti-IL15RA (Abcam).

### Image quantification.

Microscopy data were processed with multiple platforms. Tile scans were stitched using ZEN Black (Zeiss) software. Raw data were visualized and processed using Fiji (58). Automated cell segmentation, distance, and volume measurements were performed in Definiens Developer 64 using local heterogeneity segmentation to isolate CXCL12^+^ cells. Definiens rule sets for these functions were as follows:

T cell parameters: scale, α = 8; distance to neighbor, *d* = 30; and mean intensity difference to neighborhood (MDN) threshold:

(Equation 1)
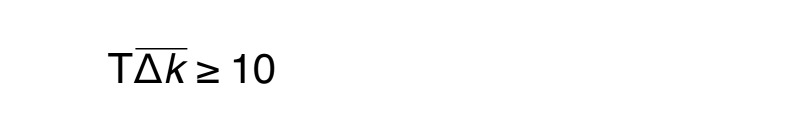


CXCL12 parameters: scale, α = 8; distance to neighbor, *d* = 30; and MDN threshold:

(Equation 2)
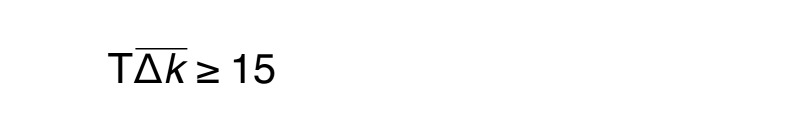


Distance measurements from this segmentation were performed as described previously (59). Cell tracking over time was analyzed using Imaris (Bitplane).

### Sample preparation for gene expression analysis.

RNA was extracted using the RNeasy Micro Kit (QIAGEN) following the manufacturer’s protocol. RNA yield, quality, and integrity were evaluated using the RNA 6000 Pico kit on an Agilent 2100 Bioanalyzer (Agilent Technologies). *Cxcl12* mRNA expression in cell suspensions from the BM was determined as previously described (55). Amplified cDNA was prepared from total RNA with an Ovation Pico WTA System V2 kit (NuGEN) for fragmentation and labeling using the Encore Biotin Module kit (NuGEN), according to kit instructions, and then hybridized onto GeneChip Mouse Gene 2.0 ST arrays (Affymetrix).

### Microarray analysis.

Hybridized arrays were scanned with a GeneChip 3000 7G scanner (Affymetrix) and the image data processed to generate .cel files. Expression Console Software version 1.4.1 (Affymetrix) was used to generate quality control statistics for each sample; only the samples that passed quality control were included in the analysis. Raw sample expression signals were background-subtracted and quantile-normalized, and the probe-level data were summarized using the Robust Multi-array Average algorithm (60, 61) implemented in the oligo BioConductor R package (62). Transcripts identified through multiple probes were collapsed based on maximum expression values using the CollapseDataset module of GenePattern software (Broad Institute) (63).

### Differential gene expression.

The limma BioConductor R package was used to perform analyses of gene differential expression, using an empirical Bayes moderated *t* statistic corrected for multiple-hypothesis testing using the Benjamini-Hochberg procedure, with a cutoff of ANOVA *P* value ≤0.01, and an absolute fold-change cutoff of ≥1.5.

### Gene set enrichment analysis.

Gene set enrichment analysis (GSEA) was performed using the GSEA software with the gene sets derived from the Reactome pathways database collected in the Molecular Signatures Database (MSigDB v5.1, Broad Institute), and the gene sets identified by Best et al. (42), Yang et al. (43), and Hurton et al. (44).

### Statistics.

Apart from microarray data, which were analyzed with the aforementioned programs and methodologies, statistical analysis was performed using GraphPad Prism version 6.00 for Mac OsX (GraphPad Software). Summary data are shown as either box-and-whisker graphs (showing median, 25th, and 75th centiles and minimum/maximum values) or means ± SD and individual replicate data. Significance was assessed using a 2-tailed Mann-Whitney *U* test or a 2-tailed Wilcoxon’s signed-rank sum test for paired comparisons of nonparametric data. Survival curve comparison was performed using the log-rank Mantel-Cox test. *P* less than or equal to 0.05 was taken to indicate a significant difference between groups.

### Study approval.

All procedures were conducted in accordance with the United Kingdom Home Office Animals (Scientific Procedure) Act of 1986, and were approved by the Animal Welfare and Ethical Review Bodies at UCL (Royal Free Campus, London, United Kingdom) and Imperial College London (South Kensington Campus, London, United Kingdom).

## Author contributions

ABK, BC, PSS, CP, CLC, and RC were responsible for the concept and design of the study, the development of the methodology, the analysis and interpretation of the data, and the writing and review of the manuscript. ST, RK, JG, PV, MZ, SG, CF, SGA, MA, AH, BF, ZRO, and TKM were involved in the development of the methodology and in the acquisition/interpretation of data. CLB, HS, and EM contributed to the development of the methodology, and the writing and review of the manuscript.

## Supplementary Material

Supplemental data

Supplemental Table 1

Supplemental Table 2

Supplemental Video 1

Supplemental Video 2

Supplemental Video 3

Supplemental Video 4

## Version 1 02/27/2018

In-Press Preview

## Version 2 04/09/2018

Electronic publication

## Version 3 05/01/2018

Print issue publication
