# Supplementary material for: Redirection to the bone marrow improves T cell persistence and antitumor functions
Source: J Clin Invest. 2018 Apr 9;128(5):2010–24. doi: 10.1172/JCI97454 (PMC5919805; doi:10.1172/JCI97454)
Supplement: Supplemental data [file jci-128-97454-s001.pdf]

Supplemental Data

Supplemental Figure 1 compares CXCR4 expression in untreated $CD8^+$ T cells, following activation with anti-CD3/CD28 beads and 3d following transduction. Supplemental Figure 2 shows the increase in local CXCL12 levels in the BM after irradiation. Supplemental Figure 3 shows staining of both endothelial and non-endothelial cells by Intravenous anti-CXCL12. Supplemental Figure 4 shows the competition profile of OT-I T^{CXCR4} and $T^{Control}$ in peripheral blood over time. Supplemental Figure 5 shows the absolute numbers of $CD62L^{high}$ and $CD62L^{low}$ cells within Ag-activated OT-I T^{CXCR4} and OT-I $T^{Control}$ populations. Supplemental Figure 6 shows greater retention of CD62L by OT-I T^{CXCR4} following vaccination with peptide-pulsed DC given intravenously. Supplemental Figure 7 shows the proliferative response to antigen of OT-I T^{CXCR4} and OT-I $T^{Control}$ following prime-boost vaccination. Supplemental Figure 8 shows the anti-tumor activity of B6-derived T^{CXCR4} following syngeneic BMT. Supplemental Table 1, included in a separate Excel file, lists the differentially expressed genes between resting memory OT-I T^{CXCR4} and OT-I $T^{Control}$. Supplemental Table 2, included in a separate Excel file, lists the Reactome pathways enriched in resting memory OT-I T^{CXCR4} and OT-I $T^{Control}$ by GSEA.

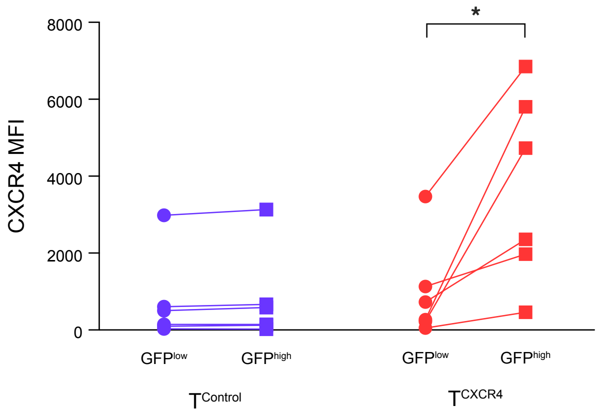

Supplemental Figure 1: Summary data showing median fluorescence index (MFI) for CXCR4 expression in GFP$^-$ and GFP$^+$ cells 3d following transduction with control *Gfp* or *Cxcr4-Gfp* vectors. Statistical comparison performed by the Wilcoxon signed rank test against a hypothetical ratio of 1.0 (indicated by the dotted line) *$p \leq 0.05$.

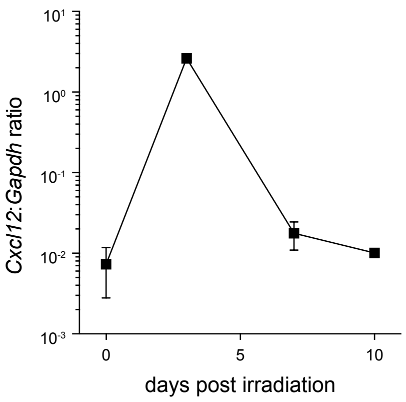

Supplemental Figure 2: Irradiation increases local CXCL12 levels in the BM. Quantitative RT-PCR was used to determine expression of *Cxcl12* mRNA at timed intervals following 5.5 Gy irradiation. Graph shows mean ±SD *Cxcl12* mRNA in relation to a *Gapdh* loading control.

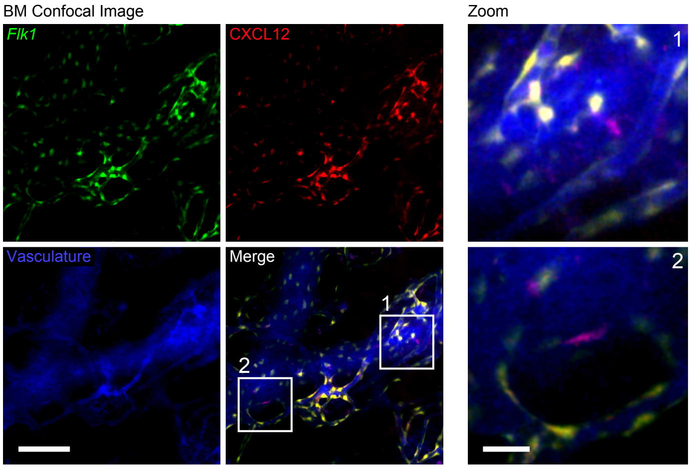

Supplemental Figure 3: Intravenous anti-CXCL12 stains both endothelial and non-endothelial cells. Intra-vital confocal calvarial imaging was performed on *Flk1-Gfp* reporter mice. Montage and high magnification insets are shown following intravenous injection of anti-CXCL12-PE (red) and Cy5-dextran to identify vasculature (blue). Yellow identifies endothelial staining of CXCL12 and purple identifies non-endothelial staining. Scale bars indicate 100 µm (montage) and 20 µm (high magnification).

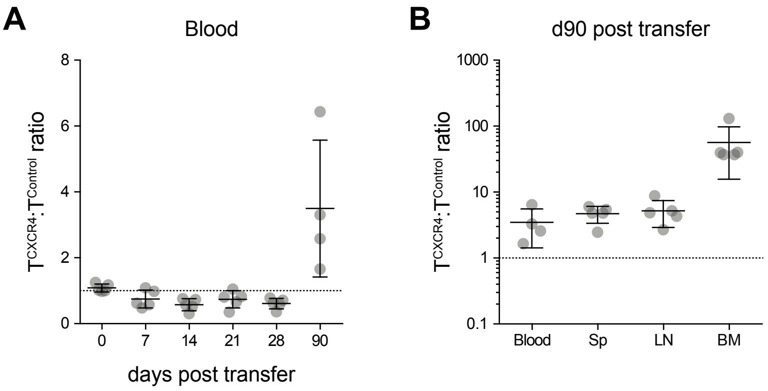

Supplemental Figure 4: Competition of OT-I T^{CXCR4} and TControl in peripheral blood. Sub-lethally irradiated syngeneic B6 recipients received intravenous injection of equal mixtures of OT-I T^{CXCR4} and $T^{Control}$ identifiable by distinct congenic markers and were then vaccinated by intra-peritoneal injection with BM-derived dendritic cells loaded with relevant peptide (SIINFEKL) followed by determination of relative numbers in peripheral blood at timed intervals. (**A**) Graph shows OT-I T^{CXCR4}:OT-I $T^{Control}$ ratio at timed intervals in peripheral blood. Statistical comparison was performed by one sample t-test against a hypothetical ratio of 1.0 (indicated by the dotted line, $p \leq 0.01$ at day 14 and day 28). (**B**) For comparison, graph shows OT-I T^{CXCR4}:OT-I $T^{Control}$ ratio in peripheral blood, spleen, lymph node and BM at day 90 in the same experiment. Note y-axis has a log scale.

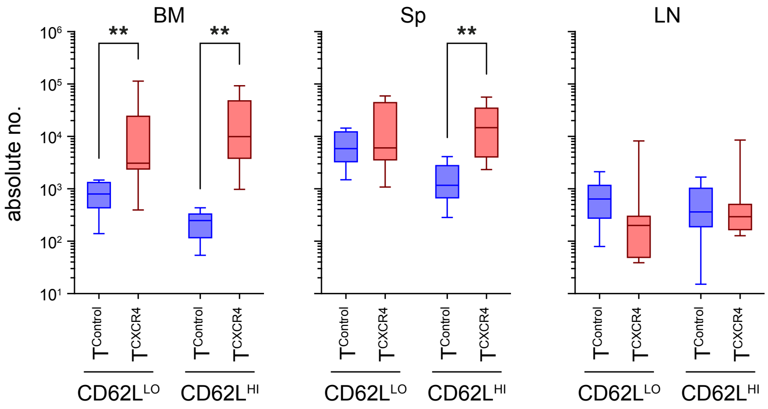

Supplemental Figure 5: Absolute numbers of CD62L^{high} and CD62L^{low} cells within Ag-activated OT-I T^{CXCR4} and OT-I T^{Control} populations. Equal numbers (5x10^{5}) of OT-I T^{CXCR4} and OT-I T^{Control} were co-injected into *Rag1ko* mice, which then underwent prime-boost vaccination with relevant SIINFEKL peptide + IFA on days 1 and 29. Graphs shown are box and whisker graphs showing absolute numbers of CD62L^{high} and CD62L^{low} OT-I T^{Control} and OT-I T^{CXCR4} on day 36 (n=9, derived from 4 independent experiments). Statistical comparisons were made by Wilcoxon matched-pairs signed rank test **$p \leq 0.01$.

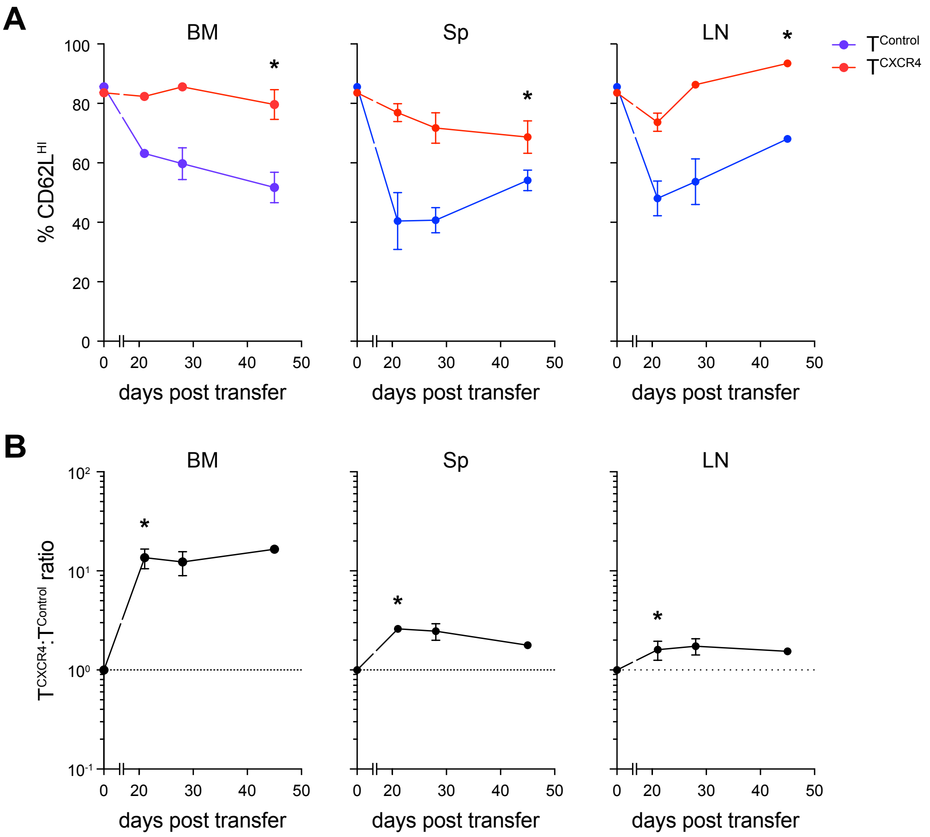

Supplemental Figure 6: OT-I T^{CXCR4} show greater retention of CD62L following vaccination with peptide-pulsed DC given intravenously. (**A**) Equal mixtures of OT-I T^{CXCR4} and $T^{Control}$ identifiable by distinct congenic markers were injected into sub-lethally irradiated syngeneic B6 recipients and vaccinated with mature $CD11c^{+}$ splenic DC loaded with relevant peptide (SIINFEKL) at 3 weeks followed by evaluation of the response. Summary data is shown for %$CD62L^{+}$ for OT-I $T^{Control}$ (blue circles) and OT-I T^{CXCR4} (red circles) in BM, Sp and LN at timed intervals (n=5 6/time point). Statistical comparisons were made by Wilcoxon matched-pairs signed rank test. Error bars denote SD. *$p \leq 0.05$. (**B**) Summary graphs indicate mean ±SD for OT-I T^{CXCR4}:OT-I $T^{Control}$ ratio at timed intervals at each site. Data are pooled from two independent experiments (n=5-6/timepoint). Statistical comparison was performed by the Wilcoxon signed rank test against a hypothetical ratio of 1.0 (indicated by the dotted line) *$p \leq 0.05$.

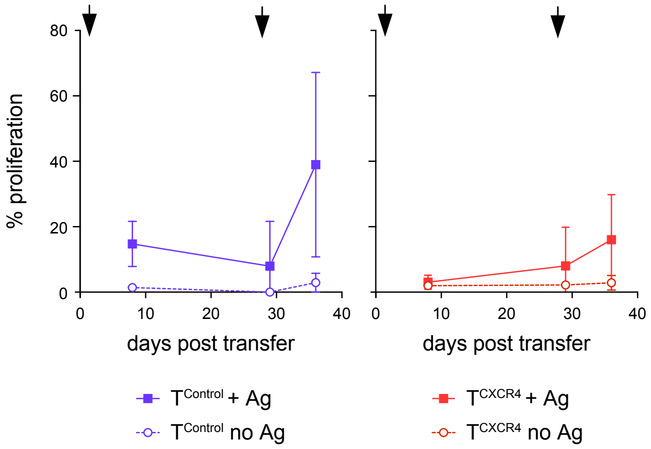

Supplemental Figure 7: Proliferative response of OT-I T^{CXCR4} to antigen is blunted following prime-boost vaccination. Equal numbers ($5x10^5$) of OT-I T^{CXCR4} (red) and OT-I $T^{Control}$ (blue) were co-injected into *Rag1ko* mice, which then underwent prime-boost vaccination with relevant SIINFEKL peptide + IFA on days 1 and 29. 5-ethynyl-2'-deoxyuridine was administered to mice by intra-peritoneal injection at timed intervals following vaccination and its incorporation evaluated 24h later. BM was harvested on day 8 (n=4/group), day 29 (n=3 Ag; n=1 no Ag) and day 36 (n=9 Ag; n=3 no Ag).

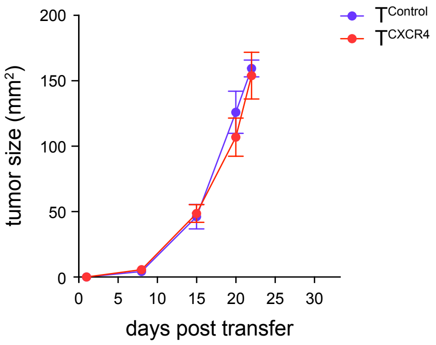

Supplemental Figure 8: B6-derived T^{CXCR4} do not demonstrate superior anti-tumor activity following syngeneic BMT. Experimental design was adapted from Figure 7A with transduced T cells being transferred to B6→B6 syngeneic BMT recipients. X-y graph shows mean ±SD A20 tumor size following transfer of $CD8^+$ $T^{Control}$ (blue) or T^{CXCR4} (red).

Supplemental Videos 1-4. Intra-vital confocal calvarial imaging of transduced T cells (green) was performed 8 weeks post injection of $T^{Control}$ and T^{CXCR4} into separate *Rag1ko* mice. Real time imaging was performed following intravenous injection of anti-CXCL12-PE (red) and Cy5-dextran to identify vasculature (blue). Data shown are videos representative of behaviour of T^{CXCR4} (Videos 1 and 2) and $T^{Control}$ (Videos 3 and 4); all videos are 30 minutes in duration.

Supplemental Table 1, included in a separate Excel file. List of the differentially expressed genes between resting memory OT-I T^{CXCR4} and OT-I $T^{Control}$ (absolute fold-change ≥1.5; ANOVA *p*-value ≤0.01).

Supplemental Table 2, included in a separate Excel file. List of Reactome pathways enriched in resting memory OT-I T^{CXCR4} and OT-I $T^{Control}$ by GSEA.
